# In vitro antifungal activity of different components of *Centratherum anthelminticum* and *Ocimum sanctum* seed oils and their synergism against oral pathogenic fungi

**DOI:** 10.15171/joddd.2016.015

**Published:** 2016-06-15

**Authors:** Aparna H Gopalkrishna, Seshagiri M, Sunil Muddaiah, Shashidara R

**Affiliations:** ^1^Reader, Department of Oral Pathology, Coorg Institute of Dental Sciences, Virajpet, Karnataka, India; ^2^Professor and Head, Department of Biochemistry, Coorg Institute of Dental Sciences, Virajpet, Karnataka, India; ^3^Professor and Head, Department of Orthodontics, Coorg Institute of Dental Sciences, Virajpet, Karnataka, India; ^4^Professor and Head, Department of Oral Pathology, Coorg Institute of Dental Sciences, Virajpet, Karnataka, India

**Keywords:** Antifungal, *Centratherum anthelminticum*, *Candida*, *Ocimum sanctum*, seed oil

## Abstract

***Background.*** Opportunistic fungal infections like candidiasis are common in the oral cavity. In recent years *Candida* species have shown resistance against a number of synthetic drugs. This study assessed the antifungal activity of *Centratherum anthelminticum* and *Ocimum sanctum* seed oils against six common pathogenic *Candida* strains. Synergistic activity of the major oil components was also studied.

***Methods.*** Antifungal activity of *Centratherum anthelminticum* and *Ocimum sanctum* seed oils were tested against six oral fungal pathogens, *Candida albicans* ATCC 90028, *Candida krusei* 6258, *Candida tropicalis* 13803, *Candida parapsilosis*22019, *Candida glabrata* 90030 and *Candida dubliniensis* MYA 646, by disc diffusion and broth microdilution methods to determine the diameter of inhibition zone (DIZ) and minimum inhibitory concentration (MIC), respectively. The oil was extracted using Soxhlet apparatus from seeds subjected to columnchromatography (CC) and thin layer chromatography (TLC) and major components were separated and quantified.

***Results.*** All the six *Candida* strains showed growth inhibition to a variable degree when tested with both seed oils. Both seed oils showed antifungal activity. For *Centratherum anthelminticum* seed oil maximum DIZ at 7 μL was recorded at 75.7 mm for *Candida albicans* ATCC 90028, and the least DIZ was 45.7 mm for *Candida dubliniensis* MYA 646. For *Ocimum sanctum* seed oil maximum DIZ at 7 μL was 61.0 mm for* Candida krusei* ATCC 6258 and the least DIZ was 46.7 mm for *Candida tropicalis* ATCC 13803. The mixtures of phospholipids and unsaponifiable matter exhibitedMIC values at 1.25 μL for both oils, whereas neutral lipids fraction and unsaponifiable matter exhibited similar MIC at 2.5 μL against *Candida albicans* and *Candida krusei*.

***Conclusion.****Centratherum anthelminticum* and* Ocimum sanctumseed* oils exhibited strong antifungal activity against six different species of *Candida* and this may be attributed to various active components in the oil and their synergistic activity.

## Introduction

The human oral cavity offers a unique and favorable environment for microbial growth.^1^Oral pathogenic fungi such as *Candida* frequently cause opportunistic infections in immunocompromised individuals, leading to morbidity and mortality.^2^*Candida* species are also known to colonize various prostheses, including dentures and implants. They can thus cause varying degrees of denture stomatitis in diabetics.^3-6^

Increasing resistance of pathogenic fungi to available antifungal agents like polyenes and azoles has highlighted the need for novel antifungal drugs with fewer side effects for effective management of candidiasis.^7,8^In the recent years, owing to its natural origin and fewer known side effects, herbal medicine is being used more frequently. There has been an increase in interest in antifungal activity of oils as they are rich in biologically active nutraceutical components.^9^A survey of literature reveals that essential oils are a mixture of simple and complex compounds, and their antimicrobial activity is well documented.^10^

*Centratherum anthelminticum*, also known as *Vernonia anthelmintica* and *Conyza anthelmintica*, is a member of *Asteraceae* family (contains more than 1000 species) of flowering plants. In India it is popularly known as bitter/black cumin or kalijiri. The seeds have a hot sharp taste and the seed extract is widely used in traditional medicine as an astringent, to cure ulcers, as an anthelmintic, in skin diseases such as leukoderma. More recently, some biologically active components isolated and identified from seeds of *C. anthelminticum* have been shown to be effective in treating breast cancer and vitiligo.^11,12^

Holy Basil in English or “Tulsi” in Hindi is scientifically known as *Ocimum sanctum.* Various parts of this plant are traditionally used in treating rheumatism, bronchitis and pyrexia. Its antioxidant properties, immunological and cardio-protective effects^13^ and antimicrobial properties have also been documented.^14^

The activity of major oil components and their synergistic activity on *Candida* have not been studied; hence this in vitro study was designed to evaluate the efficacy and activity of two different seed oils and their different components against six *Candida* strains.

## Methods

The study protocol was approved by the Institutional Review Board at Coorg Institute of Dental Sciences. This study did not involve the use of any animals or human data or tissues, and thus, an ethics approval was not required.

### Solvent extraction of total lipids

The seeds were hand-sorted to remove stones and other plant debris. The finally selected seeds were dried again in the sunlight and finely ground (a particle size of 1 to 2 mm); Soxhlet apparatus was used to extract oils with petroleum ether at a boiling point of 40‒60°C for 24 hours. The total lipids recovered were weighed and stored in chloroform at 4°C for further analysis. All the chemicals required for the present work were obtained from SD Fine Chemicals Limited, Mumbai.

### Preparation of fungal inoculums

Inhibitory activity of two oils were tested against six Candida species; *Candida albicans* ATCC 90028 (*C. albicans)*, *Candida krusei* ATCC 6258 (*C. krusei*), *Candida tropicalis* ATCC 13803 (*C. tropicalis*), *Candida parapsilosis* ATCC 22019 (*C. parapsilosis*), *Candida glabrata* ATCC 90030 (*C. glabrata*) and *C. dubliniensis* MYA 646 (*C. dubliniensis*), obtained from, the Faculty of Dentistry, University of Hong Kong.

Disc diffusion method was used to study antifungal activity. Test was performed in accordance with CLSI guidelines, 2004^15^ and using Mueller–Hinton Agar supplemented with 2% glucose and 0.5 µg/mL methylene blue dye on 90 mm diameter petri plates. The inoculum density was adjusted to the turbidity of 0.5 McFarland standard (equivalent to 1‒5×10^6^CFU/mL) at 530 nm with sterile saline. 5µl of fluconazole at a concentration of 25 µg/5 µL prepared in sterile distilled water and was used as controls. The diameter of inhibition zone (DIZ) was measured after 24hr of incubation at 35‒37°C. All the experiments were conducted in triplicate.

### Determination of minimum inhibitory concentration (MIC)

The MIC was determined by broth microdilution method according to CLSI^16^ guidelines with little modification. The 24‒72-hr cultures of *C. albicans* and *C. krusei* were standardized according to 0.5 McFarland concentration (1‒5×10^6^CFU/mL). The antifungal tests were performed on 96-well ELISA reader plates. The compounds were dissolved in dimethylsulfoxide (DMSO) to obtain a final concentration of 0.039‒5 µL. The plates were incubated at 37°C for 24-48 h; the absorbance was measured at 610 nm. The MIC was calculated based on optical density reading.

### Column chromatography (CC) and thin layer chromatography (TLC) of lipid classes

The various major components of oils were separated by eluting with different solvent system over a glass column (20 mm dia × 400 mm) packed with activated silicic acid (70‒230 mesh, obtained from Sigma Aldrich) in chloroform.

Neutral lipids, glycolipids and phospholipids were eluted with eight times the column volume of methanol. The flow rate was set at 2 mL/minute. All the three lipid fractions obtained from CC were subjected to TLC on 60 silica gel F_254_ plates (Merck KGaA Darmstadt, Germany). The phospholipid (PL), glycolipid (GL) and neutral lipid (NL) fractions were separated by developing the silica gel F_254_ plates in a suitable solvent system.^17^

PL was identified under UV fluorescent light, GL and NL were identified by placing TLC plates in iodine chamber and further identification of GL was done by using α-naphthol. UV light was used to visualize the bands; the compounds were recovered by extraction with chloroform/methanol (2:1, v/v) after scraping it from the plate.

### Quantification of lipids

Phospholipids were estimated after being separated from TLC; individual bands were scraped and brought to reaction with hydrazine sulfate/sodium molybdate reagent at 100°C for 10 min and estimated photometrically at 650 nm according to AOCS method.^18^ Quantification of glycolipids was carried out by hexose measurement method using phenol sulfuric acid in acid-hydrolyzed lipids at 485 nm.^19^

All the three different lipid components collected from the above CC and TLC procedure were stored under chloroform at 4°C for further experiments.

### Preparation of unsaponifiable matter

To obtain the sterol content of *O. sanctum* and *C. anthelminticum* seed oil^20^, 10 g of each oil were weighed into a 500-mL Erlenmeyer flask and 60 mL of 95% ethyl alcohol and 10 mL of aqueous potassium hydroxide (26.73 M) were added to the flask along with boiling chips. The mixture was refluxed for 6 h, and cooled to room temperature. The contents of the flask were transferred to an extraction cylinder; the flask was washed repeatedly with a small volume of hot and cold water until the total volume reached 150 mL. The total mixture was transferred into 500 mL separating funnel and extracted with 60-mL portions of petroleum ether five times. All the 60-mL portions were collected in another 500-mL separating funnel and the mixture was washed with 50 mL of 10% ethyl alcohol solution twice. The washed ether extract obtained from the above procedure was transferred into a 1000-mL RB flask; ether was evaporated on a steam bath and care was taken to leave a small quantity of ether in the RB flask and stored until further analysis at 4°C.

### Statistical analysis

The values of DIZ were expressed as means ± standard errors of means (SEM) and analyzed with SPSS 18.0 using one-way ANOVA and Dunnet’s t-test to compare the differences between the oils. Differences between the groups were considered significant at ^a^*P* < 0.05 and ^b^*P* < 0.01 levels.

## Results

The two tested oils and components of oils exhibited strong antifungal activity. The results obtained by disc diffusion assay are presented in Tables 1 and 2. DIZ (in mm) of all the six *Candida* species for *C. anthelminticum* seed oil was between 5 and 7 µL, ranging from 60.00 to 75.7, 53.3 to 73.7, 45.7 to 57.00, 59.00 to 69.3, 54.7 to 67.00 and 44.7 to 45.6 mm for *C. albicans*, *C. krusei*, *C. tropicalis*, *C. parapsilosis*,* C. glabrata* and *C. dubliniensis*, respectively. For *Ocimum sanctum* DIZ for all *Candida* test strains for 5 to 7 µL of oil ranged from 42.00 to 48.3, 64.30 to 61.00, 45.7 to 46.7, 54.0 to 52.0, 51.00 to 62.00 and 51.7 to 50.00 mm for all the above six *Candida* species, respectively. By increasing the concentration of the oil, there was a gradual increase in the inhibitory effect of *Centratherum anthelminticum* seed oil in comparison to that of *O. sanctum* seed oil. All the concentrations of tested oils showed significant inhibition when compared with standard drug fluconazole at 25 µg/5 µL. There was no growth beyond 7 µL for both oils.

**Table 1:** Antifungal activity of *Centratherum anthelminticum* seed oil at concentration of 1 to 7 µL

**Concentration**	**Diameter of inhibition zone (mm/µL)**					
	**A**	**K**	**T**	**P**	**G**	**D**
**FC**	24.0 ± 1.00	0.0 ± 0.0	36.0 ± 1.15	28.0 ± 1.15	29.0 ± 1.15	0.0 ± 0.0
**1**	40.0 ± 2.89^b^	40.0 ± 2.52^b^	23.3 ± 0.33	25.7 ± 0.66	23.3 ± 1.86	28.7 ± 4.33^b^
**2**	50.7 ± 2.91^b^	42.7 ± 1.76^b^	40.3 ± 2.19	36.0 ± 0.0	33.3 ± 4.18	31.3 ± 4.67^b^
**3**	55.7 ± 4.67^b^	45.3 ± 0.33^b^	37.0 ± 3.00	31.3 ± 15.7	43.3 ± 0.88^b^	32.3 ± 0.33^b^
**4**	63.7 ± 1.86^b^	51.3 ± 1.20^b^	40.7 ± 0.88	53.3 ± 2.40	52.0 ± 3.0^b^	38.7 ± 0.33^b^
**5**	60.0 ± 3.21^b^	53.3 ± 2.19^b^	45.7 ± 1.33^b^	59.0 ± 2.65^a^	54.7 ± 0.88^b^	44.7 ± 1.45^b^
**6**	66.7 ± 1.20^b^	62.0 ± 2.89^b^	51.3 ± 1.45^b^	59.0 ± 5.57^a^	62.7 ± 1.76^b^	46.3 ± 0.33^b^
**7**	75.7 ± 4.33^b^	73.7 ± 0.88^b^	57.0 ± 0.0^b^	69.3 ± 3.48^b^	67.0 ± 1.00^b^	45.7 ± 1.33^b^

Values are mean ± SEM (standard error of mean) ^a^*P* < 0.05 and ^b^*P* < 0.01 when compared with Fluconazole (FC), *Candida albicans* (A), *Candida krusei* (K), *Candida tropicalis* (T), *Candida parapsilosis* (P), *Candida glabrata* (G), *Candida dubliniensis* D.

**Table 2:** Antifungal activity of Ocimum sanctum seed oil at concentrations of 1 to 7 μL

**Concentration**	**Diameter of inhibition zone (mm/µl)**					
	**A**	**K**	**T**	**P**	**G**	**D**
**FC**	20.3 ± 3.84	0.0 ± 0.0	36.0 ± 1.15	28.0 ± 1.15	30.0 ± 0.0	0.0 ± 0.0
**1**	36.0 ± 2.08	43.3 ± 1.45^b^	26.0 ± 3.06	27.7 ± 3.38	22.3 ± 0.66	24.0 ± 2.65^b^
**2**	29.3 ± 4.33	45.0 ± 2.65^b^	32.3 ± 2.19	23.3 ± 13.0	25.7 ± 2.33	26.7 ± 2.19^b^
**3**	39.3 ± 0.66^b^	54.0 ± 1.15^b^	35.3 ± 3.53	34.0 ± 3.79	38.3 ± 1.45	34.7 ± 3.38^b^
**4**	39.7 ± 3.93^b^	62.3 ± 2.91^b^	40.7 ± 1.86	42.7 ± 2.67	42.7 ± 2.67^b^	>34.7 ± 0.88^b^
**5**	42.0 ± 3.21^b^	64.3 ± 2.85^b^	45.7 ± 2.19	54.0 ± 5.57^a^	51.0 ± 2.00^b^	51.7 ± 10.4^b^
**6**	45.3 ± 2.40^b^	70.0 ± 2.08^b^	46.0 ± 2.31	56.0 ± 3.06^a^	63.3 ± 4.37^b^	49.0 ± 2.89^b^
**7**	48.3 ± 1.76^b^	61.0 ± 2.08^b^	46.7 ± 2.85^a^	52.0 ± 2.52^a^	62.0 ± 1.00^b^	50.0 ± 0.0^b^

Values are mean ± SEM (standard error of mean) ^a^*P* < 0.05 and ^b^*P* < 0.01 when compared with Fluconazole (FC), *Candida albicans* (A), *Candida krusei* (K), *Candida tropicalis* (T), *Candida parapsilosis* (P), *Candida glabrata* (G), *Candida dubliniensis* D.

The antifungal activity of components from both the oils and their mixtures are presented in Tables 3 and 4. DIZ and MIC for two fungal pathogens *C. albicans* and *C. krusei* were selected and tested. When lipid components of both seed oils were added individually and as a mixture to the fungal culture, all the lipid components of *C. anthelminticum* exhibited significant activity when compared to standard drug as well as *O. sanctum* oil components. The MIC values ranged from 1.25 to 2.5 µL for both oil components. The mixtures of phospholipids and unsaponifiable matter exhibited significant activity at 1.25 µL for both oils, whereas neutral lipids fraction and unsaponifiable matter exhibited similar activity at 2.5 µL. The quantity of different components of both oils and unsaponifiable matters (g%/kg) are given in Table 5.

**Table 3:** Diameter of inhibition zones in mm of different oil components and mixture of oil components

**Fungal Strains**	***Ocimum sanctum***			***Centratherum anthelminticum***		
	**NL** **2 µL/3µL**	**Mixture of PL and UM (1:1)** **2 µL/3 µL**	**UM** **2 µL/3 µL**	**NL** **2 µL/3 µL**	**Mixture of PL and UM (1:1)** **2 µL/3 µL**	**UM** **2 µL/3 µL**
***Candida albicans***	50.00/CI	30.00/40.20	40.30/50.50	30.50/CI	CI/CI	CI/CI
***Candida krusei***	40.50/60.20	30.00/60.80	50.50/60.00	CI/CI	CI/CI	CI/CI

CI- complete inhibition, NL- neutral lipids, PL- phospholipids, UM- unsaponifiable matter

**Table 4:** Minimum Inhibitory Concentration (MIC) of different oil components and mixture of oil components

**Fungal strains**	***Ocimum sanctum l μ/mL***			***Centratherum anthelminticum l μ/mL***		
	**NL**	**Mixture of PL amp; UM (1:1)**	**UM**	**NL**	**Mixture PL amp; UM (1:1)**	**UM**
***Candida albicans***	2.5	1.25	2.5	2.5	1.25	2.5
***Candida krusei***	2.5	2.5	2.5	2.5	1.25	2.5

NL- neutral lipids, PL- phospholipids, UM- unsaponifiable matter

**Table 5:** Different components of oil (%) present in total lipids

**Plant material**	**Neutral lipids**	**Phospholipids**	**Glycolipids**	**Unsaponifiable matter g%/kg**
***Centratherum anthelminticum seed oil***	95.1	3.1	1.8	7.2
***Ocimum sanctum seed oil***	96.00	1.6	1.9	6.8

## Discussion

*Candida* species may not be harmful to healthy humans; however, in immunocompromised patients, illnesses, after repeatedly being treated with broad-spectrum antibiotics and in old age, may contribute to the increased incidence of focal mucosal as well as life-threatening systemic infections.^21^ The incidence of oral and systemic fungal infections has increased in recent years. A consequent rise in the mortality rate has resulted in a greater need for appropriate, more effective and safer alternatives to synthetic antifungal agents.^22-24^

*C. albicans* is the most common causative organism in *Candida*-associated infections but several other *Candida* species including *C. dubliniensis, C. glabrata, C. krusei, C. parapsilosis* and *C. tropicalis* have also been associated with the same condition.^25^

In the present investigation seed oils and their different components were tested against oral fungal pathogens, exhibiting significantly better inhibition than the standard drug fluconazole. The results showed that both oils tested inhibited the growth of all the *Candida* species, including the highly resistant *C. krusei*. At concentrations higher than 7 µL both oils also exhibited a fungicidal action. It was also observed that *C. anthelminticum* seed oil and its different components exhibited maximum activity at a very low concentration (1.25 µL) when compared to *O. sanctum* (2.50 µL) seed oil. The results for both seed oils also clearly indicated that antifungal activity of the oils is dose-dependent. Earlier findings on antimicrobial activity of seed oil and plant extracts have shown a dose-dependent increase in activity.^26,27^ We reported the MIC values of *C. albicans* and *C. krusei* only as the former is a common oral pathogen and the latter a highly resistant strain.

When the individual components of the oils were tested, it was observed that the mixture of phospholipids and unsaponifiable matter at a ratio of 1:1 of both oils exhibited activity, whereas *C. anthelminticum* seed oil mixture exhibited complete inhibition at lower concentrations compared with *O. sanctum*. No antifungal activity was seen from the glycolipids obtained from the seeds.

Recently around nine biologically active highly oxygenated stigmastane-type steroids have been isolated from aerial parts of *Vernonia anthelmintica.*^12^ When the highly oxygenated stigmastane-type steroid compounds were examined on estrogen biosynthesis in human ovarian granulosa-like KGN cells, there was an increase in 17-β-estradiol biosynthesis in a dose-dependent manner. Compounds containing such Δ^7^sterol structure have been shown to have antimicrobial activity.^28^ An earlier study has also reported on the antifungal activity of methanolic extract of *C. anthelminticum* seeds against *C. albicans*, albeit with mild to moderate results as opposed to the results of the present study*.*^29^

A study conducted on 2019 patients with candidemia revealed that *Candida* species comprised up to 54.4%, with *C. albicans* being the most frequently isolated species. It was also observed that even patients with single episode of candidemia had a mortality rate of 35.2%, with the highest rate among patients with* C. krusei* candidemia and the lowest among patients with *C. parapsilosis* candidemia.^30^

The antifungal properties of most of the essential oils are attributed to the presence of highly oxygenated sesquiterpenes and some minor components which have synergistic activity with active compounds.^31^The results of this study clearly indicated that phytosterols present in the unsaponifiable matter of *C. anthelminticum* seed oil have high biologic activity. When phospholipid fractions are added to unsaponifiable matter at a ratio of 1:1, the activity of sterols or other molecules may increase. The synergistic effect of pure components in higher concentrations might be responsible for strong antimicrobial property of oil components. Previous phytochemical studies on *C. anthelminticum* seeds has revealed the presence of over 120 compounds which exhibited various biological activities.^11^

*O. sanctum* has a is highly complex chemical composition. The chemical components of leaf, volatile oil, as well as other parts of the plant and their antimicrobial activity on different pathogens are well documented.^13,14^We showed that *C. anthelminticum* oil contains more biologically active molecules than *O. sanctum* seed oil. This variation in the quantity of active components of *O. sanctum* and *C. anthelminticum* seed oils, especially phospholipids and unsaponifiable matter**,** might have resulted in the different degrees of antifungal activity.

Previous studies on different types of oils have suggested that the antimicrobial activity depends on the quantity of active components present in these oils. The quantity and quality of these components depend on various factors such as climate, soil, water availability and other seasonal variations.^32,33^Components of essential oil, their solubility in water and their ability to penetrate the microbial cell walls is directly related to the capability of altering the lipid bilayer membrane; this may alter the functions of membrane and membrane-bound enzymes, which may cause alterations in the synthesis of cell wall components.^4,34^Addition of phospholipids may increase the penetration of biologically active molecules through the lipid bilayer membrane of fungal cell, which may inhibit fungal growth.^35^ This study also showed that addition of major components in a proper proportion may have better activity than individual components. However, differences in the activity of phytochemicals depend on the quantity and quality of compounds.^2,19^

Previous research work indicates that the antimicrobial activity of oils is predominantly due to the presence of oxygenated terpenoids and to a lesser extent due to hydrocarbons. It was also observed that the combination of major and minor components could result in synergistic effect.^36^ Chloroform fraction of *C. anthelminticum* seeds was reported to have antioxidant property. Vernodalin, identified as the active compound, possesses antineoplastic properties and induces cell cycle arrest and apoptosis.^37^

## Conclusion

*C. anthelminticum* and *O. sanctum* seed oils exhibited potent antifungal activity against the tested oral fungal pathogens. This may be due to the synergistic activity of different active components and their respective quantities present in these oils. However, further studies are required to identify and purify other active ingredients and evaluate their synergistic activities for future use in preventing and treating oral candidiasis.

## Acknowledgment

The authors would like to thank Dr. Lakshman Samaranayake, former Dean, HKUSD, for providing the *Candida* strains.

## Authors’ contributions

The study was conceptualized by AHG and SEM; SEM was involved in extraction of components from the seeds and chromatography. SHR was involved along with the AHG and SEM in carrying out the antifungal activity tests and preparation of the initial draft of the article. Statistical analysis and refinement of the article was carried out by SM. All the authors were involved in preparing the final draft of the article.

## Funding

The study was self-funded.

## Competing interests

The authors declare that they have no competing interests with regards to authorship and/or publication of this paper.

## Ethics approval

Not applicable.
